# The Road to Hell Is Paved With Good Intentions: How Common Practices in Scale Construction Hurt Validity

**DOI:** 10.1177/10731911221124846

**Published:** 2022-09-29

**Authors:** Diana Steger, Kristin Jankowsky, Ulrich Schroeders, Oliver Wilhelm

**Affiliations:** 1 University of Kassel, Germany; 2 Ulm University, Germany

**Keywords:** scale construction, ant colony optimization, construct coverage, validity, health knowledge

## Abstract

Sound scale construction is pivotal to the measurement of psychological constructs. Common item sampling procedures emphasize aspects of reliability to the disadvantage of aspects of validity, which are less tangible. We use a health knowledge test as an example to demonstrate how item sampling strategies that focus on either factor saturation or construct coverage influence scale composition and demonstrate how to find a trade-off between these two opposing needs. More specifically, we compile three 75-item health knowledge scales using *Ant Colony Optimization*, a metaheuristic algorithm that is inspired by the foraging behavior of ants, to optimize factor saturation, construct coverage, or a compromise of both. We demonstrate that our approach is well suited to balance out construct coverage and factor saturation when constructing a health knowledge test. Finally, we discuss conceptual problems with the modeling of declarative knowledge and provide recommendations for the assessment of health knowledge.

Textbooks on psychological measurement usually list reliability and validity as primary goals of test construction (e.g., Coaley, 2010). Although reliability comprises different aspects such as stability over time, equivalence across parallel test forms, and internal consistency, in practice the concept of reliability is often condensed to the overall consistency of a measure. Different coefficients to assess reliability, among others Cronbach’s alpha and McDonald’s omega, have been repeatedly discussed and compared in the psychometric literature (Green et al., 1977; McNeish, 2018; Schmitt, 1996; Sijtsma, 2009). In contrast, the term “validity” conveys many different meanings (Borsboom, 2006) and some of them are difficult to quantify. For example, content validity is often just asserted, seemingly justified on a theoretical basis or determined using expert ratings (Clark & Watson, 2019; Sireci, 1998). In educational assessment, some fields suit more objective approaches to numerically quantifying the content validity of performance measures (e.g., Vanauer et al., 2022). Similarly, domains with finite sets of items such as adjectives within the lexical approach of personality assessment (Goldberg et al., 2006) could legitimately claim content validity, although sampling from such sets is rarely done at random but instead is tailored to reproducing the big five personality factors. However, the overarching majority of psychological measures suffers from a lack of quantifiable content validity and this may be one reason why reliability is often the main focus of item selection in scale construction (Kruyen et al., 2013).

The relationship between reliability and validity is often understood as interdependent, for example, in the principle that reliability is a necessary, but not sufficient, prerequisite for validity. Conceptually, such simplifications might cause substantial problems. Maximizing reliability—using indicators that focus on internal consistency such as selecting items based on their item-total-correlation or factor saturation—may hamper validity through a homogenization of the item pool. In extreme cases, parts of the construct may systematically be neglected, resulting in decreased content validity (Loevinger, 1954). In the present paper, we illustrate how different item sampling strategies that focus on maximizing either consistency or construct coverage affect the properties of the resulting scale. We further demonstrate how both criteria can be integrated simultaneously to construct scales that are both reliable and valid.

To this end, we apply *Ant Colony Optimization* (ACO; Olaru et al., 2019; Schroeders et al., 2016a)—an advanced item sampling approach that allows for the simultaneous optimization of multiple selection criteria—to select items from a broad pool of knowledge questions. We chose health knowledge as an example due to its hierarchical and multi-dimensional structure which makes it sensitive to issues of item sampling. As the internal structure of any knowledge test is contingent on the level of abstraction (Steger et al., 2019) and the predictive accuracy may strongly depend on the specific items set (Schroeders et al., 2016b, 2021), scale construction in knowledge assessment is an ideal showcase for demonstrating the versatility of metaheuristics such as ACO in finding a compromise between homogenization and diversification of an item set.

## Aspects of Reliability

There are plenty of guidelines and practical examples on sound psychometric scale development (e.g., Benson, 1998; Boateng et al., 2018; Clark & Watson, 1995, 2019; Morgado et al., 2018; Zickar, 2020). Although both reliability and validity are fundamentally important for psychological measurement, item selection in test construction often focuses on specific aspects of reliability (e.g., Cronbach’s α if item is deleted or part-whole corrected item-total correlations; Kruyen et al., 2013). Similarly, in their systematic review on common practices when reporting psychological measures, Flake et al. (2017, p. 370) found “that validity evidence of existing and author-developed scales was lacking, with coefficient α often being the only psychometric evidence reported.”

Within the predominant reflective measurement approach, it is desirable to provide collections of indicators that deliver substantial relationships with the factors onto which they are regressed. In the simplest and most prevalent case, one tends to construct scales that are precise and unidimensional measures of a single target construct (Strauss & Smith, 2009). We argue that this overemphasis on internal consistency has several drawbacks. Cronbach’s α—as the most prominent representative of these indices—is often misinterpreted (Sijtsma, 2009), for example as an indicator of unidimensionality. In addition, Cronbach’s α is likely to be biased due to violated assumptions (McNeish, 2018) and also of limited utility in longer scales (i.e., > 40 items; Clark & Watson, 2019; Cortina, 1993). However, there is a more fundamental problem which also concerns the use of other indicators of reliability that come with less strict assumptions (such as *factor saturation*, which can be conceptualized in a factor analytical framework as the ratio of the variance explained by items compared to the total variance of a factor): Selecting items that have much in common (i.e., highly interrelated items) also leads to scales that are artificially narrowed in content. This phenomenon has been termed the *attenuation paradox* (Loevinger, 1954) where an extreme increase in reliability leads to a less diverse item pool, which most likely represents only a narrower part of the construct. It has been argued that such a one-sided item selection procedure results in a measure that only partially reflects the target construct (Clark & Watson, 1995; Clifton, 2020) because it is exclusively optimized for high item-intercorrelations rather than offering an empirical representation of a theoretical construct (Borgstede, 2019; Buntins et al., 2016). Nonetheless, selecting highly homogeneous item sets enjoys popularity if the scale in question is narrowly circumscribed and consists of a modest number of items.

## Aspects of Validity

Validity has become somewhat of a “catch-all” term for complex psychometric questions (Borsboom, 2006) and the importance of the concept of validity is in stark contrast to its vague and elusive definition. Over time, more and more aspects of validity were introduced into the literature, leading to a complex and diverse collection of psychometric approaches and theories—including the simple statement that a scale is valid when it “really measures what it purports to measure” (Kelley, 1927, p. 14), as well as when the emphasis is placed on embedding the construct in a nomological net (Cronbach & Meehl, 1955), on ontology, reference, and causality (Borsboom et al., 2004), or on prediction (Shmueli, 2010; Yarkoni & Westfall, 2017).

Most concepts of validity are interwoven and partially overlapping (Pike, 1992) and can be related to Loevinger’s (1957) three components of construct validity: the substantive component, the structural component, and the external component. First, the *substantive component* emphasizes the content of the test (Sireci, 1998). A thorough literature review or an expert survey should promote the development of a clear construct definition and a broad, comprehensive item pool (Clark & Watson, 1995, 2019) so that the scale adequately covers the relevant aspects of a construct. Loevinger’s (1957) substantive component of validity is thus highly concerned with issues of construct coverage and item content—concepts that are also central to the concept of content validity (Haynes et al., 1995). Second, the *structural component* deals with questions of dimensionality and inter-item relationships. Here, it is evaluated whether the empirical structure corresponds to the theoretically assumed structure of the construct—aspects that are sometimes labeled *dimensional* or *factorial* validity. Finally, the *external component* is concerned with the scale’s relationships to other constructs (i.e., convergent or discriminant validity; Campbell & Fiske, 1959) or its ability to predict various outcomes (i.e., criterion validity or predictive power). Some of these terms are connected, which is why they need to be considered in conjunction: For example, if relevant parts of the construct are neglected (i.e., if content validity is impaired), this presumably also leads to a decrease in the predictive power (i.e., criterion validity decreases) because then the measure no longer corresponds to the intended level of generalization—which is a prerequisite for estimating an unbiased relationship between two constructs according to Wittmann’s (1988) adapted version of Brunswik’s (1955)*lens model*.

The three components of validity can be hard to disentangle conceptually and hard to model empirically, given that aspects of validity are often hard to quantify. For example, construct validity in its original conceptualization (Cronbach & Meehl, 1955) builds upon the relationships to other scales and one expects to find high correlations with measures of the same or similar constructs (convergent validity), and low correlations with measures of different constructs (discriminant validity). However, one cannot rely on general recommendations on the magnitude of these correlations—appropriate cutoffs depend on several aspects (reliability of the measure, characteristics of the study sample, etc.). The problem is exacerbated for content validity, for which validation approaches are mostly qualitative in nature and based on construct definitions and expert judgments, thereby making content evaluation especially hard for constructs that lack a clear definition (Haynes et al., 1995).

Suppose that an almost exhaustive item pool for a specific construct has been established. The question remains as to how to construct a scale that maintains construct coverage. In cases where the sub-facets of the construct are well-described in the literature and empirically supported in previous studies, one might select items according to their allocation to these sub-facets. This strategy however requires profound knowledge of the construct. In cases where there is little prior knowledge, one might take into account item-intercorrelations (as advocated by Clark & Watson, 1995, in the tradition of Loevinger, 1957). More precisely, items should not be selected with the exclusive goal of maximizing item-intercorrelations, but items with moderate correlations should be selected to retain coverage and to avoid artificial narrowing of the construct (Briggs & Cheek, 1986; Clark & Watson, 1995). This approach thus focuses not only on the average item-intercorrelations but also on the distribution of correlations. The magnitude of item-intercorrelations may determine the level of abstraction with which we measure a given construct: By choosing items that are highly inter-correlated, the measure we generate shows higher internal consistency but measures only a narrow facet of a construct. In contrast, by choosing items that are only moderately inter-correlated, the measure we generate is a broader representation of the construct. Ideally, we seek to balance these two contradicting criteria: We want to select items that are correlated highly enough to allow precise measurement of the construct while at the same time limiting the magnitude of item-intercorrelations so that scales are not artificially narrowed in terms of coverage—or, put differently, without impairing content validity. Counterbalancing these criteria requires the use of sophisticated item sampling approaches.

## Using Meta-Heuristics in Item Selection

Traditional approaches to item selection often apply a sequential procedure in which individual items are evaluated based on a single statistical value such as the item-total correlation (or some other index that indicates the homogeneity of a scale such as factor loadings; for an overview, see Kruyen et al., 2013). However, this evaluation is based on the relationship between the indicators and the initial item sample rather than the set of indicators that constitutes the final scale. As a result, traditional procedures optimize a scale sequentially—that is, an item that is removed from the sample cannot be reconsidered at later stages of test compilation. This approach might lead to a biased sample because removing items from the item pool will lead to a change in the parameters that are used for item selection (Olaru et al., 2015).

To avoid biases due to this sequential procedure, it is better to use meta-heuristic item sampling approaches (e.g., Schroeders et al., 2016a). One representative of these meta-heuristics is ACO (Olaru et al., 2015, 2019; Schroeders et al., 2016a). To date, ACO has been applied to derive psychometrically sound short scales in various fields of psychological assessments (e.g., Janssen et al., 2017; Kerber et al., 2021; Olaru & Danner, 2021). For example, its usage permits the minimization of measurement variance across age while simultaneously retaining model fit and construct coverage (e.g., Olaru & Jankowsky, 2022) as well as the optimization of correlations with covariates while also accounting for reliability, item difficulty, and model fit (Schroeders et al., 2016a).

The ACO algorithm is inspired by the foraging behavior of ants: Analogous to ants “communicating” best routes to food sources by leaving pheromone trails, ACO uses virtual pheromone values to identify optimal item combinations. In a first iteration, random item sets are evaluated according to some pre-defined criteria. The metaheuristic “learns” by iteratively increasing the selection probability of positively evaluated item sets. Across several iterations of selecting and evaluating item combinations, a close-to-optimal solution can be found (Olaru et al., 2019). In addition to avoiding sequence effects in item selection and the ability to include several criteria simultaneously, ACO is also less computationally demanding in comparison to evaluating all possible item combinations. ACO is thus a promising technique for the present study because it allows including multiple criteria simultaneously—even if they might be in an opposing relationship to each other such as factor saturation and construct coverage.

## The Present Study

**Figure 1.**
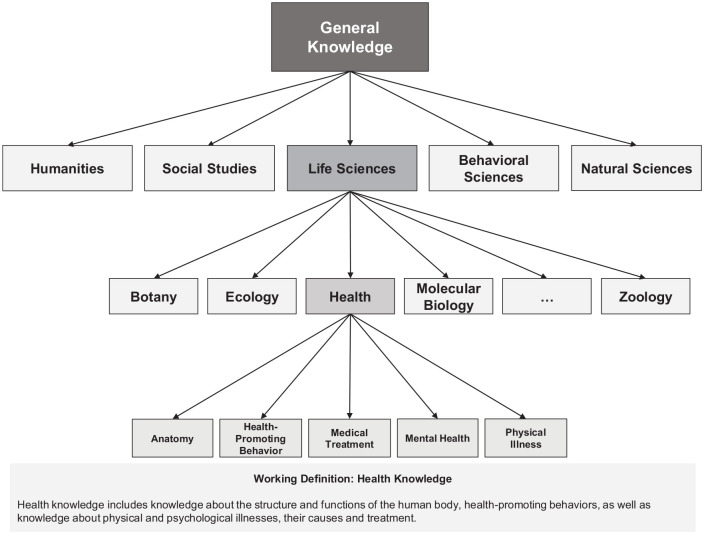
Health Knowledge in the Hierarchy of General Knowledge.

In the present study, we chose the measurement of health knowledge as an example to illustrate the consequences of different item sampling approaches. Although health knowledge is an important facet of various conceptualizations of health literacy (e.g., Chin et al., 2011; Ownby et al., 2014), few attempts have been made so far to measure health knowledge broadly in a non-specialized population (Beier & Ackerman, 2003). Here, we broadly define health knowledge as knowledge about the structure and functions of the human body, health-promoting behaviors, as well as knowledge about physical and psychological illnesses, including their causes and treatments. In Figure 1, we illustrate the hierarchical structure of declarative knowledge in relation to our concept of health knowledge.

Using health knowledge as an example is especially promising because knowledge assessment in general is not trivial: Declarative knowledge is a hierarchical and multidimensional construct (Steger et al., 2019) and the internal structure hinges largely on item sampling. This stresses the importance of content validity, rendering health knowledge (as a broad facet of declarative knowledge) an interesting case for demonstrating the effects of different item sampling approaches. Moreover, due to the breadth of knowledge, the impact of artificial narrowing of the scale content might be particularly large (Stadler et al., 2021) if item selection is focused on optimizing reliability. In turn, if broad indicators of declarative knowledge are used, estimates of internal consistency or factor saturation may turn out poor. For example, Rolfhus and Ackerman (1999) assessed the psychometric properties of 20 broad academic knowledge tests and despite a large amount of items, estimates of Cronbachs α varied largely, ranging from .56 to .91. Similarly, Steger et al. (2019) assessed 22 broad knowledge scales and reported expected *a posteriori* estimate (EAP) reliabilities ranging from .63 to .84. In both cases, the estimates might be systematically inflated due to the high number of items per scale, and shorter scales will likely result in lower reliability estimates.

To demonstrate the effects of the different item selection strategies, we compile three versions of a 75-item health knowledge scale: One with a focus on factor saturation as an indicator of reliability, one with a focus on construct coverage as an indicator of content validity, and finally, one where we try to balance out the potentially conflicting criteria by combining factor saturation and construct coverage to construct a scale that is both reliable *and* valid. We compare the three item selection strategies with regard to their ability to meet the respective pre-specified criteria and compare the psychometric properties of the resulting scales on both item and scale levels. Finally, we report the item overlap of the three scales against the background of the different item selection strategies.

## Method

To make the analyses transparent and reproducible, we provide all materials (i.e., data, syntax, and supplemental tables and figures) online at https://osf.io/8pds2/?

### Design and Participants

To illustrate the different item selection procedures, we reanalyzed data collected via a mobile quiz app from October 2016 to January 2020 (Steger et al., 2019). This knowledge test was part of a larger study assessing cognitive abilities which was approved by the Ethics Committee of Ulm University. Participants (total *N* = 6,737) worked on a total of 4,050 questions from 34 knowledge domains designed to broadly assess knowledge. All knowledge items used multiple-choice format and were presented in sets of 27 questions per round. Participants were free to choose how many questions they wanted to answer akin to the *Synthetic Aperture Personality Assessment* technique (Condon & Revelle, 2014), resulting in data with large proportions of missingness (Revelle et al., 2017). We based our analysis on a subsample of items with health-related content. Participants were included in the analysis if they answered at least one third of the selected health knowledge items. In total, 520 participants (56.2% female) with a mean age of 40 years (*SD* = 16 years) were included in the analysis. Regarding their educational background, 4.81% of the participants reported having a degree from a vocational-track school, 17.69% reported having a degree from an intermediate-track school, 31.92% reported having a degree from an academic-track school, and 43.27% reported having a university degree (2.31% reported having no degree).

### Item Selection

**Figure 2.**
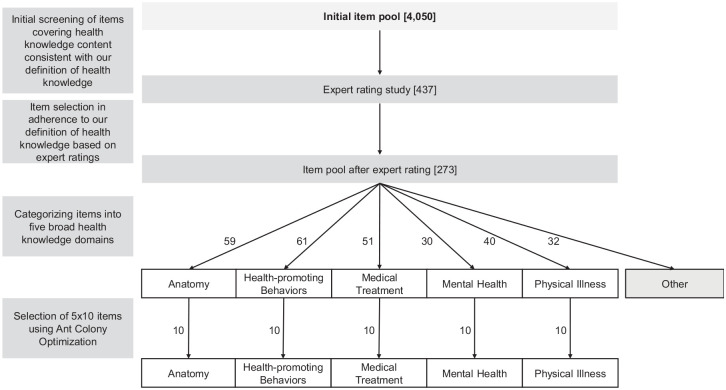
Item Selection Procedure. *Note*. The final item pool for the present analysis comprised 241 items because items that could not be categorized unanimously into one of the five domains (“Other”) were excluded from the analysis.

Figure 2 depicts the various steps applied to compile the 75-item measures of health knowledge. First, we identified broad knowledge domains (e.g., *Medicine, Health*, or *Psychology*) or sub-domains (e.g., *anatomy* [*Biology*], *food* [*Nutrition*], or *medical engineering* [*Technology*]) with potentially health-related content from the original item pool (Steger et al., 2019), resulting in a reduced item pool of 437 items. These items were included in an expert rating study. Thirty-nine participants (69% female) with a mean age of 35 years (*SD* = 12 years) with a vocational background in a health-related field (e.g., medicine, psychology, or nursing) were asked to indicate whether item content is health-related according to our definition of health knowledge. The reduced item pool after expert ratings consisted of 273 items for which at least 70% of the raters approved content in line with our definition of health knowledge. The cut-off for expert agreement was chosen to sample health knowledge as broadly as possible without including irrelevant items in the analysis. Finally, items were grouped into five broad domains of health knowledge based on our definition of health knowledge by three independent raters (Fleiss’ κ = .89). We excluded all items (*n* = 32) that were not assigned unanimously to one of the five knowledge domains by the three raters, resulting in a final item pool of 241 health knowledge items that served as a basis for the subsequent analyses.

### Item-Sampling Using Ant Colony Optimization

For the 75-item health knowledge scales, we specified a model with five correlated factors consisting of 15 items each. Parameter estimations were based on a *weighted least squares means and variance adjusted* (WLSMV) procedure with pairwise complete data. A root mean square error of approximation (RMSEA) < .05, and a comparative fit index (CFI) > .95 were considered as indicators of good model fit (Hu & Bentler, 1999). ACO was run with three different optimization functions: For the item sampling procedure that focused on the optimization of construct coverage (ACO_Cov_), we tried to minimize the averaged Fisher-transformed item-intercorrelations for each of the five factors, using a cut-off of *M_r_* = .20. We also aimed for a selection in which the standard deviation of item correlations was similar to those in the initial item pool. We thus minimized the difference between the dispersion of the long and the abbreviated version for each factor. In doing so, we tried to find a proxy for optimizing construct coverage. For the item sampling procedure in which we focused on the optimization of consistency (ACO_Con_), we calculated ω_cat_ for dichotomous data (Flora, 2020; Green & Yang, 2009). Since breadth in content is characteristic of domains of declarative knowledge, we used an empirical approach to derive domain-specific cut-offs for the optimization of factor saturation rather than using arbitrary cut-offs that might fail to account for the peculiarity of declarative knowledge. For this purpose, we estimated the factor saturation of 10,000 randomly drawn item sets for each health knowledge domain (see Table S1). For each domain, we used the 99th percentile as the optimization criterion, resulting in the following cut-off values: ω_anatomy_ > .58, ω_behavior_ > .60, ω_illness_ > .71, ω_mental_ > .63, ω_treatment_ > .60. For the balanced version (ACO_Cov+Con_), we combined construct coverage and consistency by including all of the above-described criteria. For more information on the three optimization functions, see the Appendix. Problematic models (i.e., models resulting in errors or warnings) were not included in the optimization process. As ACO is a probabilistic approach that may result in different psychometrically sound solutions across several runs, we carried out the item selections ten times with different seeds. In the following, we only present the best solution of these 10 runs based on the overall pheromone value.

## Results

In Table 1, we juxtaposed the specified optimization criteria for the three ACO runs with the results of the three health knowledge scales. Overall, a clear picture emerged for all three resulting health knowledge scales: First, in two ACO runs—namely ACO_Cov_ and ACO_Con_—it was possible to find an item set that met the pre-specified criteria. For ACO_Cov+Con_, in which the seemingly contradictory criteria were included, all but one criterion were met. Second, both ACO_Cov_ and ACO_Con_ failed to meet the criterion that was not specified in their respective optimization function. Furthermore, the mean item-intercorrelations within factors were by far the lowest for ACO_Cov_; they were higher in ACO_Cov+Con_ and highest in ACO_Con_ (see Table S2 for details). A similar pattern emerged for the standard deviations of the item-intercorrelations: In ACO_Cov_, the standard deviations were, as intended, essentially identical to those of the final health knowledge item pool. ACO_Cov+Con_ showed slightly more deviation from the original values and ACO_Con_ showed the largest deviation. For factor saturation and mean factor loadings, this pattern was reversed in that factor saturation and mean loadings were the highest in ACO_Con_, lower in ACO_Cov+Con_ (with the exception of *Mental Health*, for which ACO_Cov+Con_ had slightly higher factor saturation and mean loadings than ACO_Con_) and the lowest in ACO_Cov_. To sum up, both ACO_Cov_ and ACO_Con_ clearly exceeded the specified cut-offs. More importantly, these cut-offs could also largely be met in the balanced solution despite the seemingly contradictory results.

**Table 1.** Comparison of the Specified Criteria and the Results of the Health Knowledge Scales.

Optimization criteria			Construct coverage	Balanced solution	Consistency
Model fit	CFI	Criterion	>.95	>.95	>.95
		Result	1.00	.96	.96
	RMSEA	Criterion	<.05	<.05	<.05
		Result	.00	.01	.01
Item-intercorrelation	*M*_r_	Criterion	<.20	<.20	*Not included*
		Result	.05	.18	.28
	Δ*SD*_r_	Criterion	.00	.00	*Not included*
		Result	<.01	.01	−.03
Factor saturation	ω_anatomy_	Criterion	*Not included*	.58	.58
		Result	.26	.66	.70
	ω_behavior_	Criterion	*Not included*	.60	.60
		Result	.36	.66	.69
	ω_treatment_	Criterion	*Not included*	.60	.60
		Result	.22	.65	.71
	ω_mental_	Criterion	*Not included*	.63	.63
		Result	.34	.67	.66
	ω_illness_	Criterion	*Not included*	.71	.71
		Result	.35	.47	.77

*Note.* CFI = comparative fit index; RMSEA = root mean square error of approximation; ω = factor saturation, *M*_r_ = mean item-intercorrelations, SD_r_ = Standard deviation of item-intercorrelations.

In addition, the effects of homogenization versus diversification on item-level can also be transferred to scale-level. When computing unidimensional models of health knowledge using parcels based on 15 items per domain as indicators (see Table S3 for more information on the measurement models), the same pattern emerges: Factor saturation of the general health knowledge factor is by far the lowest for the version that focused on construct coverage only (ω = .38), higher for the solution with balanced criteria (ω = .63), and highest for the version that focused on internal consistency only (ω = .69).

The differences in the construct coverage between the selected scales is also apparent in the item content of the respective versions: Focusing on consistency during item selection, 12 out of 15 items sampled from the domain of *Health-Promoting Behaviors* assessed nutritional knowledge, whereas other topics such as effects of drug consumption or personal hygiene were not selected. In fact, three out of ten items had “vitamin C” as their correct response, illustrating the redundancy of item content. In contrast, by focusing on construct coverage, items in the domain of *Health-Promoting Behaviors* covered a much broader range of topics, including nutrition, fitness, effects of drug consumption, or personal hygiene. For the balanced version, the trade-off between factor saturation and construct coverage is also reflected in item content: As in the version that focused solely on factor saturation, the majority of items sampled from the domain of *Health-Promoting Behaviors* stresses nutritional knowledge. However, the scope of the nutritional knowledge is broader and includes knowledge about micro- and macronutrients, general dietary recommendations, and specific diets. In addition, the other items covered topics such as drinking or disease prevention.

**Figure 3.**
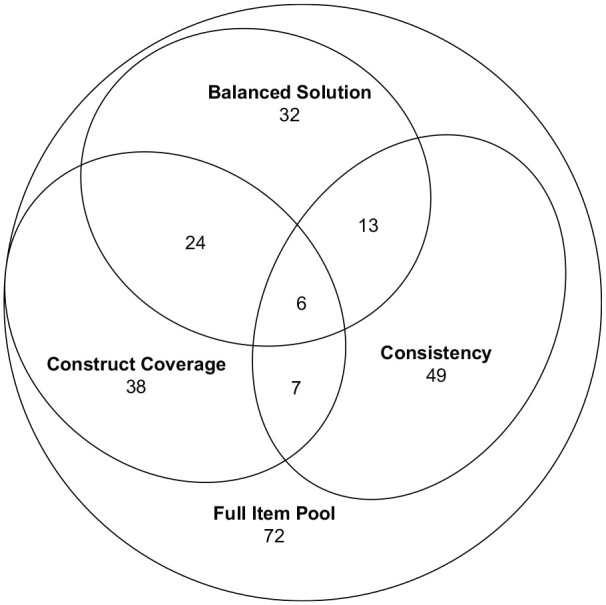
Venn Diagram of Selected Items for the Three Different Item Sampling Strategies.

Finally, we display item overlap between the three item sets in Figure 3. The health knowledge scale that was compiled using ACO_Con_ represented the most unique item sample, whereas the balanced version had the largest overlap with the two other item sets, mirroring the similarities of the optimization functions: The items sets that were sampled with opposing criteria (construct coverage vs. factor saturation) have fewer items in common as compared to their overlap with the item sample that included both criteria. Overall, there were six items included in all three resulting scales. The item overlap between ACO_Cov_ and ACO_Cov+Con_ (30 items) is larger than one would expect it to be if the items were drawn completely randomly (expected overlap^1^: 23 items), while the item overlap between ACO_Cov+Con_ and ACO_Con_ (19 items), as well as the overlap between ACO_Cov_ and ACO_Con_ (five items), are smaller than expected by chance.

## Discussion

Although psychological measures should be both reliable and valid, in practice validity is often neglected in test construction in favor of internal consistency (Clifton, 2020; Flake et al., 2017). Optimizing internal consistency is comparatively easy to achieve whereas optimizing aspects of validity is harder. The common practice of solely focusing on internal consistency, however, impacts not only the validity of the measure but also ultimately the validity of scientific results (Flake & Fried, 2020). To this end, we demonstrated how optimizing different selection criteria resulted in item sets that fundamentally differ in their psychometric characteristics. An item selection algorithm that focuses on factor saturation counteracts construct coverage, which resulted in scales that achieved high factor saturation at the cost of being redundant in terms of content. In contrast, emphasizing construct coverage impedes factor saturation to a considerable extent, which also affects the interpretability of measurement models (Heene et al., 2011). We also demonstrated how to balance between factor saturation and construct coverage using ant colony optimization, resulting in a scale that meets both the psychometric requirements of adequate model fit and factor saturation while retaining the breadth of content.

Obviously, the results with the present item sets might not easily generalize across fields of psychological assessment. In addition, the optimal setup and weighting of different optimization criteria is an empirical question (for an introduction on how this could be practically achieved, see Olaru et al., 2019). Moreover, there might be fields in which it is simply not possible to find an appropriate trade-off between internal consistency and construct coverage. First, if the resulting scale is consistent but it is not possible to retain coverage of the measured trait, the latent variable is narrower than intended. In this case, it is advisable to reconsider the construct label to highlight the reduced scope of the measurement (Clifton, 2020). Alternatively, researchers could revise and extend the scope of the item pool and start over. Second, if it is not possible to retain consistency but the resulting scale has appropriate construct coverage, different modeling approaches with less strict requirements might be suitable. Such a strategy of test development (which is akin to what psychometric textbooks discuss as external test construction) could rely on formative measurement models. One goal of such scales can be to optimally separate groups based on the prediction of an outcome. Third, if it is impossible to meet the demands of both consistency and coverage, the measure does not reflect a psychological trait or ability as a disposition that is stable across time and situations.

### ACO—A New Tool in the Methodological Toolbox

The present results again evidenced the versatility of ACO in integrating a variety of (potentially conflicting) optimization criteria simultaneously, thereby adding to the literature that used ACO in item selection and short scale construction (e.g., Janssen et al., 2017; Kerber et al., 2021; Olaru et al., 2019; Schroeders et al., 2016b). In this paper, we argue for a compromise between consistency and coverage in item sampling. Other aspects of measurement such as the elimination of differential item functioning, adaptive testing, and test linking are also legitimate goals of item compilation. ACO is not limited to a specific method such as correlational analysis to target construct coverage. Other methods such as network analysis, methods of information reduction, or models of item response theory are conceivable. Apart from the methods applied, the goals pursued can also be diverse. For example, other aspects of validity may come to the fore in different contexts of test construction. Concerning convergent or discriminant validity, correlations between the scale and relevant criteria might play a role (e.g., Schroeders et al., 2016a). In the case of health knowledge, one might for instance compile a measure that is substantially related to established measures of health literacy—as for example with the *European Health Literacy Survey Questionnaire* (Sørensen et al., 2013) or the *Health Literacy Questionnaire* (Osborne et al., 2013). Concerning predictive validity, ACO can be set to favor item sets that increase the relationship to outcomes of importance. For example, for fibromyalgia patients, disease-specific health knowledge was shown to predict relevant health outcomes such as the ability to carry out everyday activities or the intensity of pain (Camerini et al., 2012). Other disease-specific health knowledge tests could be compiled to predict treatment compliance or else a general health knowledge test could serve to predict global health status in the general population. Concerning content validity, ACO might be instantiated to identify item sets that take into account experts’ ratings of cognitive operations, assumed skill sets necessary to solve an item, or prototypicality of indicators. Importantly, all more-ore-less implicit criteria that might steer test construction have to be disclosed and quantified.

Integrating ACO as a powerful new tool in the methodological toolbox of test constructors also shifts the focus in test development. If test compilation is to be delegated to an algorithm, the initial item pool from which short scales are drawn under certain constraints must be extensive and also reflect various aspects of the construct as completely as possible. For this purpose, it will be necessary to more systematically involve experts in the respective content areas. Also, the heterogeneity of the construct should be reflected in the diversity of test developers. For example, when it comes to cross-cultural research, the team of test constructors should be recruited from the participating countries as has been done in PISA (Program for International Student Assessment). As a consequence, more complex test designs (i.e., multiple matrix design, Gonzalez & Rutkowski, 2010) are necessary to administer a large number of items to test-takers without excessively increasing the individual workload. Both the *Synthetic Aperture Personality Assessment* (SAPA, Condon & Revelle, 2014; Revelle et al., 2017) and the *International Cognitive Ability Resource* (ICAR, Dworak et al., 2021) are good examples of collecting empirical data to a large item pool.

### Conceptual Challenges in Modeling Declarative Knowledge

Declarative knowledge is usually conceptualized as a broad and hierarchically structured construct for which Cattell (1971, p. 121) argued that, in adults, crystallized abilities “extend into Protean forms,” stressing its diversity, differentiation, and idiosyncrasy. Similarly, Ackerman (1996, p. 241) stated that “there are probably as many domains of knowledge as there are occupations (and nonoccupational pursuits as well).” Given this stance, few researchers attempted to analyze the factorial structure of crystallized intelligence. Instead, much of the research on crystallized abilities is built on vocabulary tests, even though knowledge tests are well-suited indicators for crystallized abilities (Schipolowski et al., 2015). Accordingly, Ackerman (2000, p. 69) pointedly described declarative knowledge as “the dark matter of intelligence”, expressing its significance in understanding cognitive functioning and how this significance contrasts with our incapacity to measure and model it adequately.

The obstacle to using fact knowledge questions more widely is not found in items. The knowledge requirements of an item are usually sufficiently evident. The obstacle instead seems to be the fuzziness of scales and factors. The scope of a test hinges upon the desired granularity as well as domain sampling and item sampling procedures (Steger et al., 2019). For example, a proxy of crystallized abilities might include items of several disparate broad knowledge areas such as the humanities, social sciences, and natural sciences. However, one can easily “zoom in” at any point in the knowledge landscape and subcategorize into domains such as the arts, music, history, and geography—or into even narrower subdomains, such as history of art, architecture, drawing, photography, and so on. In principle, the desired granularity of an assembled test need not follow discrete steps. Instead, in most instances a continuous fine-tuning of the scope seems possible. The idea of a factor space with statistical abstractions such as first-, second-, and third-order factors therefore seems somewhat simplistic and obsolete and yet we persist in using such concepts because they are easy to visualize, to understand, and to communicate.

One might argue that health knowledge is a somewhat uncomplicated domain for illustrating the consequences of trimming the content for consistency versus coverage. On one hand, we could indeed capitalize on prior work from different fields to deliver an inclusive understanding of the domain. On the other hand, knowledge assessment is less than unequivocal, that is, marking the boundaries of the knowledge domain also means excluding content that other researchers might consider essential (e.g., biopharmacological technology, for instance, by which means phages kill multi-resistant bacteria). Inevitably, applying the strategies illustrated here to other domains requires careful consideration of the scope of the construct, which entails a thorough search of the available literature with no guarantee that prior research exhausted the scope of the construct. Moreover, using the same settings as in the present case, one might face new psychometric shortcomings of the scale which necessitate modified sampling approaches. How successfully such a compromise between consistency and coverage can be made will depend on the measurement intention, the breadth and depth of the knowledge included, the size of the initial item pool, and other boundary conditions. In the present proof-of-concept, we had no additional health indicators to empirically double-check the superiority of the method against other methods. In psychological assessment, the practical utility of a measure is often the key factor. Thus, high criterion-related or incremental predictive validity of the resulting measures–which were not included as criteria in the optimization function itself–would substantiate the described procedure.

### Psychometric Challenges in Modeling Declarative Knowledge

Traditional approaches of item selection usually assume that the gold standard of scale construction is to compile a scale that is a unidimensional, precise measure of the target construct which adheres to the prerequisites of a reflective measurement model. In such models, items are indicators of a latent construct which cannot be measured directly but serve as a common cause, accounting for communality between items (Borsboom et al., 2004; Markus & Borsboom, 2013a). These assumptions might hold for items measuring fluid abilities that stem from a well-defined item pool and comprise comparatively few item attributes. It is less likely that these restrictive assumptions hold for measures of crystallized abilities (Kan et al., 2011). Formative models could serve as an alternative. In these models, test scores are not assumed to reflect a latent entity but are mere composite scores of the observed data. However, this also implies that the construct does not exist independently of the measure. Items are not considered interchangeable and the selection of indicators might rely highly on the measurement purpose, making it difficult to generalize results to other measurement occasions or to other samples. Moreover, both reflective and formative modeling approaches fall short of accurately accounting for construct-immanent variance at the item level (Mõttus, 2016; Schroeders et al., 2021), which is in focus when prediction is emphasized over explanation (Shmueli, 2010; Yarkoni & Westfall, 2017). If our goal is *predicting* an outcome accurately—without necessarily *explaining* a particular phenomenon—adhering to models that focus on aggregate levels might not be expedient. The emphasis on item-specific variance, however, is at odds with mainstream psychometric models such as confirmatory factor analysis.

Another modeling framework that might be applicable for declarative knowledge is the behavior domain theory (Markus & Borsboom, 2013a; McDonald, 2003). Instead of assuming a causal relationship between latent variables or factors on one side and items on the other, behavior domain theory assumes a sampling relationship: Constructs are seen as domains of behavior, and item responses are samples from this domain. In this case, inferences about the relationship between factors and items require generalization rather than causal inference—putting the focus on content validity and representative item sampling as a necessity for valid measurement (Markus & Borsboom, 2013b). For the assessment of declarative knowledge, this would imply that knowledge domains can be interpreted as behavior domains and item samples from these knowledge domains are samples from this behavior domain (e.g., Schipolowski et al., 2015; Steger et al., 2019).

Both assumptions might not apply for knowledge assessment. Neither the level of granularity nor the borders of neighboring domains are sufficiently evident in models of declarative knowledge. It is also unclear whether within-item multidimensionality should be admitted or penalized. At the level of factors, knowledge domains need not be mutually exclusive: Some domains evidently represent an overlap of two or more different fields (e.g., bioinformatics, philosophy of physics, or art history). Therefore, many items or—in traditional terminology—lower-order factors could be assigned only ambiguously to the next higher level. Some symptoms of these problems are model misfit, cross-loadings, or correlated residuals. We would also count the fuzziness of domains and resulting problems in communicating research results as among these issues. As behavior domain theory also requires clear-cut unidimensional measurement models for the domain of interest (Markus & Borsboom, 2013b; McDonald, 2003), they are not very likely to constitute a solution for the problems we face in measuring fact knowledge as a key component of crystallized abilities. One possible solution would be to reduce the fuzziness and purify the domains until no ambiguous indicators are left in an item sample. But would such purified domains still be content valid? Would we deem their coverage adequate? Probably not: Unlike other constructs where similar deviations from the unidimensional measurement model represent measurement error, in the present case of knowledge assessment it is mainly construct-relevant variance that is at odds with our measurement conceptions.

### Measuring Health Knowledge

Although health knowledge is essential for preventing and curing diseases as it helps individuals to communicate symptoms with health-care providers or to understand the importance of adhering to a treatment regime (Bryant, 2002; Freimuth, 1990), few attempts have been made to measure health knowledge broadly (Beier & Ackerman, 2003). Instead, most attempts to measure health knowledge either focused on specialized populations of health-care providers (Simonsen et al., 2011) or assessed only facets of health knowledge (e.g., knowledge about physical illnesses; Gellert et al., 2018; mental health knowledge; Wei et al., 2016), lower-order topics (e.g., nutrition; Parmenter & Wardle, 1999), or specific diseases (e.g., diabetes; Eigenmann et al., 2011). Also, research on health literacy often relies not on ability tests to measure health knowledge but on self-reports (Osborne et al., 2013; Sørensen et al., 2013) which might be better suited for assessing participants’ self-concept rather than their actual ability (e.g., Freund & Kasten, 2012).

In the present paper, the compilation of health knowledge tests served mostly illustrative purposes. The results clearly speak in favor of the instrument that satisfies both content coverage and factor saturation. This instrument represents a broad and psychometrically sound measure of health knowledge: We sampled from a large set of items, had experts evaluate the items’ relevance, and checked compatibility with prior work from different subfields. This approach is more eclectic and exhaustive than what is usually done in the literature. Our definition of health knowledge conceptualizes health knowledge largely overlapping with a general dimension of knowledge about the life sciences (Steger et al., 2019), including knowledge from domains of nutrition, medicine, psychology, or biology. The present approach can be deemed more inclusive than other approaches that are centered more narrowly around physical diseases and conditions (e.g., Beier & Ackerman, 2003). Accordingly, we provide the newly constructed health knowledge scale online in English and German: https://osf.io/8pds2/.

### Implications for Future Research

Constructing an instrument to measure general health knowledge might be a first step toward a better understanding of health knowledge in general and how it might be related to health outcomes. Past research often found health knowledge to be beneficial regarding several health outcomes such as levels of general functioning (e.g., Camerini et al., 2012), thereby stressing its preventive character. However, knowledge is mainly acquired through experience and hinges largely on the biographical experiences of an individual. Accordingly, it is also plausible that it is not only particularly healthy individuals who have a great deal of health knowledge but also individuals who have already suffered from diseases. It is also plausible that biographical effects differ depending on the specific diseases (Gellert et al., 2018). Future research might address the relationship between health knowledge and different health outcomes. Specifically, the different levels of abstraction of health knowledge should be juxtaposed with varying levels of abstraction in health outcomes—from broad outcomes (e.g., general well-being or general health) through more specific outcomes (e.g., days of sick leave) to very specific health outcomes (e.g., presence of specific illnesses, or treatment success). Following the idea of symmetry between predictors and criteria (Brunswik, 1955; Wittmann, 1988), more general measures of health knowledge might be better suited for predicting more general outcomes, whereas more fine-grained measures of specific topics should be better suited for predicting more specific outcomes.

In general, we advise future researchers to pay attention to construct coverage during test construction, and especially during item selection. With the present paper, we raise the question as to what extent popular measures are characterized by a lack of construct coverage, a fact that is amplified by common psychometric procedures of item selection. We present contemporary statistical tools to overcome this issue. As test developers, we recommend refocusing on the content of social science measurement, specifically in the measurement of declarative knowledge.

## Appendix

As a first criterion, we considered model fit with values for CFI > .95 and RMSEA < .05 as indications of good model fit, but without differentiating above these thresholds. Values below the specified thresholds were logit-transformed to differentiate more strongly between values close to the respective cutoff and to scale the value range between 0 and 1:

(1)
$$\varphi_{\mathrm{CFI}}=\{\begin{array}{c} \frac{1}{1+e^{100*\left(.95-\mathrm{CFI}\right)}}:CFI<.95\\ \;1:CFI\geq .95 \end{array},$$

(2)
$$\varphi_{\mathrm{RMSEA}}=\{\begin{array}{c} \frac{1}{1+e^{100*\left(.05-\mathrm{RMSEA}\right)}}:RMSEA>.05\\ \;1:RMSEA\leq .05 \end{array}.$$

As a second criterion, we considered the 99th percentile of ω as a cut-off for each respective domain (*Health-Promoting Behavior* > .60, *Anatomy* > .58, *Medical Treatment* > .60, *Mental Health* > .63, and *Physical Illness* > .71 to be sufficient:

(3)
$$\varphi_{\mathrm{Factor}\;\mathrm{Saturation}}=\frac{1}{1+e^{20*(\mathrm{cutoff}-\omega_{d})}}.$$

For the third criterion, we minimized the averaged Fisher transformation-based correlations within each of the five factors. For the average correlations, our criterion was .20:

(4)
$$\varphi_{\mathrm{cor\_M}}=\frac{1}{1+e^{20*(.20-M(\mathrm{cor}))}}.$$

In addition, we minimized the mean absolute difference between the standard deviations of item correlations within the short and long forms:

(5)
$$\varphi_{\mathrm{cor}\_\mathrm{SD}}=\left(1-\frac{1}{1+e^{100*M\left(.05-\mathrm{abs}\left(\mathrm{SD}(\mathrm{cor},\;\mathrm{short})-\mathrm{SD}(\mathrm{cor},\;\mathrm{long})\right)\right)}}\right).$$

We then averaged across *M* and *SD* of the correlations:

(6)
$$\varphi_{\mathrm{Correlation}}=\frac{\varphi_{\mathrm{cor}\_M}+\;\varphi_{\mathrm{cor}\_SD}}{2}.$$

In the three different pheromone functions (Construct Coverage, Balanced Solution, and Consistency), the respective criteria were summarized and maximized as follows:

(7)
$$\mathrm{Maximize}\;f\left(x\right)=\varphi_{\mathrm{CFI}}+\varphi_{\mathrm{RMSEA}}+\varphi_{\mathrm{Correlation}},$$

(8)
$$\begin{array}{c} \mathrm{Maximize}\;f\left(x\right)=\varphi_{\mathrm{CFI}}+\varphi_{\mathrm{RMSEA}}+2*\varphi_{\mathrm{Correlation}}+\\ \varphi_{\mathrm{Factor}\;\mathrm{Saturation}}, \end{array}$$

(9)
$$\mathrm{Maximize}\;f\left(x\right)=\varphi_{\mathrm{CFI}}+\varphi_{\mathrm{RMSEA}}+\varphi_{\mathrm{Factor}\;\mathrm{Saturation}}.$$

## Notes

1. Expected item overlap between k item sets of the size $n_{set}$ sampled at random from an item pool of the size $N_{tot}$ can be computed as follows:

$$item\;overlap=N_{tot}.{\left(\frac{n_{set}}{N_{tot}}\right)}^{k}$$
